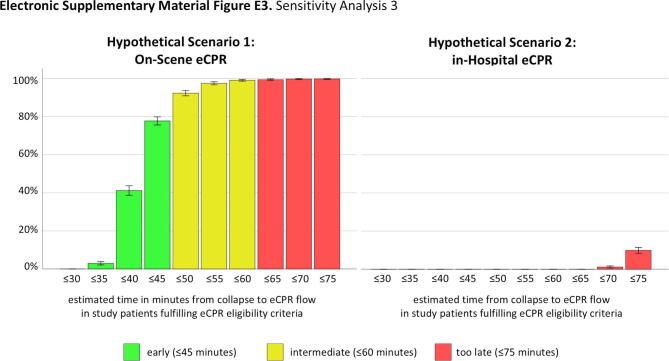# Eligibility and hypothetical time windows for eCPR in out-of-hospital cardiac arrest patients managed by a nationwide helicopter-emergency medical service: A multicentric, retrospective cohort study

**DOI:** 10.1016/j.resplu.2025.101160

**Published:** 2025-11-14

**Authors:** Romana Erblich, Matthias Noitz, Helmut Trimmel, Wolfgang Voelckel, Dinis Dos Reis Miranda, Dominik Jenny, Oliver Hunsicker, Maximilian Havlicek, Julian M. Baumkirchner, Jens Meier, Martin W. Dünser

**Affiliations:** a Department of Anesthesiology and Critical Care Medicine, Kepler University Hospital and Johannes Kepler University, Linz, Austria; b OEAMTC Flugrettung, OEAMTC, Vienna, Austria; c Department of Anaesthesiology, Emergency Medicine and Intensive Care, Hospital Wiener Neustadt, Wiener Neustadt, Austria; d Department of Anaesthesiology and Intensive Care Medicine, AUVA Trauma Centre Salzburg, Academic Teaching Hospital of the Paracelsus Medical University, Salzburg, Austria; e Department of Intensive Care, Erasmus MC University Medical Centre, Rotterdam, the Netherlands; f Helicopter Emergency Medical Services, Trauma Centre Zuid-West Nederland, Erasmus MC University Medical Centre, Rotterdam, the Netherlands; g Charité Universitätsmedizin Berlin, corporate member of Freie Universität Berlin and Humboldt-Universität zu Berlin, Department of Anaesthesiology and Intensive Care Medicine CCM/CVK, Berlin, Germany; h Department of Urology, University Hospital Zürich, Zürich, Switzerland

**Keywords:** Out-of-hospital cardiac arrest, Non-traumatic, eCPR eligibility, Time to eCPR, On scene eCPR, In-hospital eCPR

## Abstract

**Aim:**

To determine the proportion of out-of-hospital cardiac arrest (OHCA) patients, who fulfilled eligibility criteria for extracorporeal cardiopulmonary resuscitation (eCPR) on scene and were managed by a national helicopter emergency medical service (HEMS). Additionally, we compared estimated time intervals from collapse until hypothetical eCPR for the scenarios of on scene and in-hospital eCPR.

**Methods:**

This multicentre, retrospective study included 6687 non-traumatic OHCA patients (01/2010–12/2019). Time intervals from collapse until hypothetical eCPR were calculated using measured, published or estimated data.

**Results:**

ECPR eligibility criteria were fulfilled in 890 [13.3 % (95 %CI, 12.5–14.2 %)] of 6687 patients. In these patients, the estimated time from collapse to hypothetical eCPR was shorter in the on scene than the in-hospital eCPR scenario (42.2 ± 5.2 vs. 82.4 ± 6.1 min, *p* < 0.001). More patients underwent hypothetical eCPR within 35, 40, 45, 50, 55, 60, 65, 70 and 75 min after collapse in the on scene compared to the in-hospital eCPR scenario (*p* < 0.001 for all comparisons). The proportions of patients, who fulfilled eCPR eligibility criteria but could not undergo hypothetical eCPR within 60 or 75 min, were lower in the on scene compared to the in-hospital eCPR scenario [60 min: 0.9 % (95 %CI, 0.4–1.8 %) vs. 100 % (95 %CI, 99.6–100 %), *p* < 0.001; 75 min: 0.1 % (95 %CI, 0–0.6 %) vs. 87.4 % (95 %CI, 85.1–89.5 %), *p* < 0.001].

**Conclusion:**

In Austrian HEMS, one in seven OHCA patients fulfilled eCPR eligibility criteria on scene. Our estimates strongly suggest that initiation of eCPR on scene could significantly shorten low-flow times and increase the proportion of eligible OHCA patients, who receive this potentially life-saving intervention.

## Introduction

Out-of-hospital cardiac arrest (OHCA) is a devastating condition associated with limited chances of survival,^1^ even under optimal circumstances.^2^ In conjunction with conventional cardiopulmonary resuscitation, extracorporeal cardiopulmonary resuscitation (eCPR) improves survival of selected patients with OHCA.^3^, ^4^ Robust evidence to identify those patients, who might benefit from eCPR, is currently lacking. A recent systematic review found a marked variability in eCPR eligibility criteria across systems.^5^ Contemporary guidelines recommend considering eCPR in younger patients (<65–70 years) without life-limiting comorbidities, minimal no-flow times (*e.g.*, witnessed cardiac arrest and bystander cardiopulmonary resuscitation), and a known or suspected treatable cause of cardiac arrest.^6^, ^7^ So far, only few data have been published on how many OHCA patients actually fulfil these criteria on scene and would potentially qualify for eCPR.^8^

Although current guidelines recommend that extracorporeal blood flow should be established within 60 min after collapse,^6^, ^7^ clinical data indicate that the odds of survival decrease with every minute delay until initiation of eCPR.^12^, ^13^, ^14^, ^15^, ^16^ As eCPR is, in most places, a hospital-based intervention, eligible OHCA patients need to be transported to an eCPR centre while ongoing CPR is provided. The necessity for intra-arrest transport of these patients prolongs the time to establish extracorporeal blood flow and frequently results in delays from collapse to initiation of eCPR therapy exceeding 60 min, rendering patients eventually ineligible for this potentially life-saving intervention.^10^ While some centres have started to treat OHCA patients with mobile eCPR teams on scene^17^ and clinical trials on prehospital eCPR therapy are ongoing,^18^ current guidelines recommend that patients with refractory OHCA, who are suitable for eCPR, should be transported to the nearest hospital providing eCPR.^7^

In this study, we determined the proportion of subjects, who fulfilled guideline-informed eCPR eligibility criteria on scene, among 6687 OHCA patients managed by a nationwide helicopter emergency medical service (HEMS). In addition, we evaluated and compared estimated time intervals until eCPR for the hypothetical scenarios of on scene eCPR and in-hospital eCPR following intra-arrest transport.

## Materials and methods

This analysis was designed as a multicentre, retrospective, observational cohort study. It included data of patients managed by the teams of thirteen HEMS bases of the OEAMTC Air Rescue service (Christophorus Flugrettungsverein, OEAMTC, Vienna, Austria) from January 1, 2010 until December 31, 2019. The study was part of a larger research project aiming to investigate the characteristics, treatment, and outcomes of patients with OHCA managed by the OEAMTC Air Rescue service. This research project was evaluated and approved by the Ethics Committee of the Federal State of Lower Austria (GS4-EK-4/703-2020). Due to the retrospective study design, written informed consent was waived. The results of a prior analysis focusing on the outcomes of a selected group of OHCA patients have been published before.^19^ This manuscript was prepared in line with the Strengthening the Reporting of Observational Studies in Epidemiology (STROBE) guidelines.^20^

### Study setting

The OEAMTC Air Rescue service is a private organisation contractually bound to provide HEMS in Austria. During the study period, the OEAMTC Air Rescue service operated sixteen HEMS bases in all nine Austrian states. Fifteen bases operated from dawn to dusk, while one was operational for 24 h. Three bases (Vienna, Salzburg, Nenzing) were run in cooperation with local emergency medical services and did not provide medical records for this analysis because of specific data protection regulations by the three local partners. HEMS teams consisted of one prehospital emergency physician (75 % of whom were anaesthesiologists), one flight paramedic, and one pilot. The decision whether a physician-staffed rapid response car and/or a HEMS team was dispatched to a patient with OHCA was made by regional ambulance control centres. It depended on availability, accessibility, and the estimated time needed to reach the scene of OHCA.

### Patient population

The medical records of the electronic database of the OEAMTC Air Rescue service were screened for primary missions responding to patients with a confirmed OHCA. No age limits were applied. Patients with traumatic OHCA and those with signs of irreversible death (*e.g.*, livor or rigour mortis) at HEMS arrival were excluded.

### Data extraction

The following data were extracted from the electronic database for all study patients: age, sex, location where cardiac arrest occurred, witness status of cardiac arrest, bystander response, initial ECG rhythm, HEMS response time (time from dispatch alarm to landing close to the scene), presence of additional advanced critical care teams on scene before arrival of the HEMS team, return of spontaneous circulation (ROSC) at arrival of the HEMS team, time the HEMS team spent on scene, flight time to hospital (in patients with a sustained ROSC), and immediate patient outcome (no ROSC vs. sustained ROSC).

### Study goals

The primary study goal was to determine the proportion of OHCA patients, who fulfilled eCPR eligibility criteria at arrival of the HEMS team on scene. The secondary study goal was to determine and compare the estimated time intervals from collapse to eCPR for the two hypothetical scenarios of on scene eCPR or hospital-based eCPR in patients fulfilling eCPR eligibility criteria. Based on guideline recommendations to establish eCPR within 60 min of collapse,^6^, ^7^ we determined and compared the proportions of patients, who fulfilled eCPR eligibility criteria on scene but would hypothetically not undergo eCPR within 60 min of collapse between the two scenarios.

### ECPR eligibility criteria

In line with contemporary guidelines by the European Resuscitation Council^6^ and the Extracorporeal Life Support Organisation,^7^ we used the following criteria, all of which needed to be met in order to be eligible for eCPR: (1) age ≥12 years and <70 years; (2) collapse witnessed by bystanders or emergency medical staff; (3) bystander cardiopulmonary resuscitation or cardiopulmonary resuscitation by emergency medical staff (when witnessed by emergency medical staff); (4) shockable rhythm or pulseless electrical activity as the initial ECG rhythm; and (5) no sustained ROSC at HEMS arrival on scene. We chose a minimum age of 12 years to be eligible for eCPR. This pragmatic decision was based on the fact that the 50th percentile for height of children at the age of 12 years is 150 cm,^21^ which allows for cannulation of femoral vessels using standard cannulas for eCPR (*e.g.*, arterial cannula: 15–17 Fr; venous cannula: 19–25 Fr).^7^

Given the lack of a universally agreed eCPR eligibility criteria,^5^ we also conducted sensitivity analyses applying different eCPR eligibility criteria. The first sensitivity analysis applied an upper age limit of 65 years with no changes to the remaining eCPR criteria as indicated above. The second sensitivity analysis applied only shockable rhythms as the initial ECG rhythm with no changes to the remaining eCPR criteria as indicated above. The third sensitivity analysis applied all initial ECG rhythms with no changes to the remaining eCPR criteria as indicated above.

### Hypothetical eCPR scenarios and calculation of time intervals

**Fig. 1:**
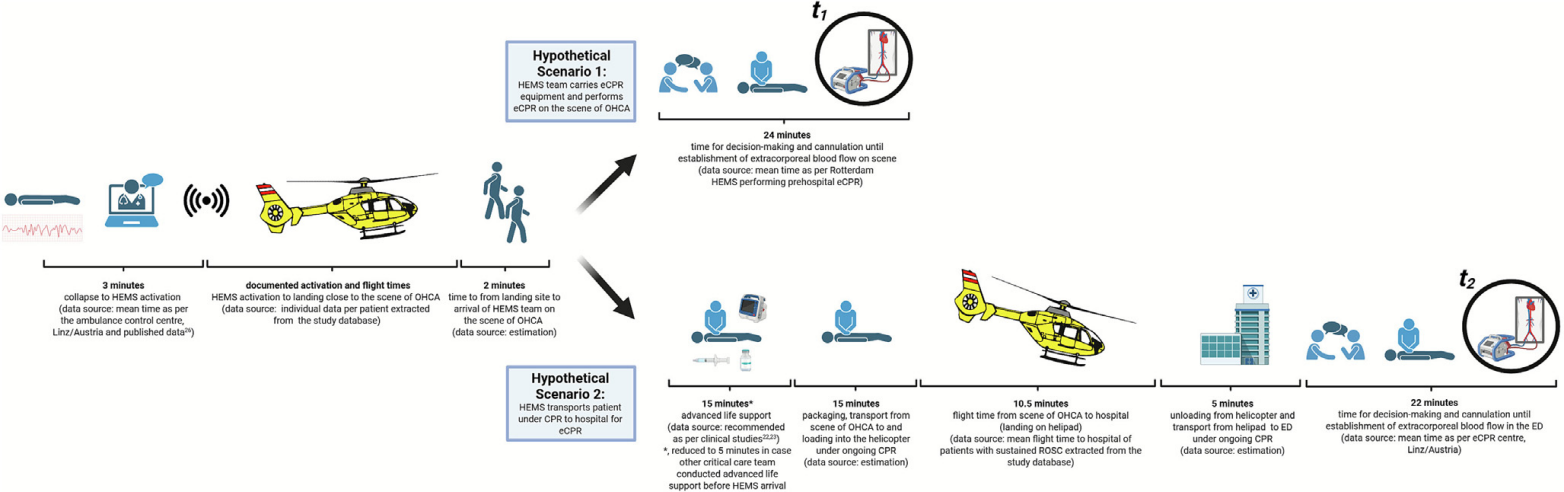
Overview of estimated time intervals from collapse to hypothetical eCPR on scene (*t*_1_) and in-hospital (*t*_2_). eCPR, extracorporeal cardiopulmonary resuscitation; HEMS, helicopter emergency medical service; OHCA, out-of-hospital cardiac arrest; ROSC, return of spontaneous circulation.

In every study patient fulfilling eCPR eligibility criteria, two hypothetical scenarios were constructed (Fig. 1). Hypothetical scenario 1 assumed that the HEMS team brought eCPR expertise and equipment to the scene enabling on scene eCPR. Hypothetical scenario 2 assumed that the patient was transported under ongoing cardiopulmonary resuscitation to the hospital for in-hospital eCPR. Time intervals from collapse until hypothetical eCPR were calculated using measured, published or estimated data as outlined below and in Fig. 1.

The time from collapse to eCPR for scenario 1 was calculated by summing up the following time intervals: time from witnessed collapse to dispatch alarm of the HEMS team (3 min; data source: mean time interval as per the ambulance control centre in Linz/Austria) plus time from HEMS team dispatch to landing close to the scene (data source: documented time intervals extracted from the study database) plus time to overcome the distance from the landing site to the scene of OHCA (2 min; data source: estimation based on clinical experience) plus time to initiate eCPR on scene (24 min; data source: mean time interval as per Rotterdam HEMS, a service performing on scene eCPR).

The time from collapse to eCPR for scenario 2 was calculated by summing up the following time intervals: time from witnessed collapse to dispatch alarm of the HEMS team (3 min; data source: mean time interval as per the ambulance control centre in Linz/Austria) plus time from HEMS team dispatch to landing close to the scene (documented individual time intervals extracted from the database) plus time to overcome the distance from the landing site to the scene of OHCA (2 min; data source: estimation based on clinical experience) plus advanced life support on scene (15 min; data source: results of clinical studies^22^, ^23^; in case another advanced critical care time had arrived at the scene before the HEMS team and had already provided advanced life support, this time was reduced to 5 min) plus time to package, transport to and load the patient in the helicopter under ongoing cardiopulmonary resuscitation (15 min; data source: estimation based on clinical experience) plus flight time to the hospital (10.5 min; data source: mean flight time in patients achieving a sustained ROSC as extracted from the study database) plus time from helicopter landing at the hospital to arrival in the emergency department including time for helicopter engine shutdown, unloading and transport from the helipad to the emergency department while providing ongoing cardiopulmonary resuscitation (5 min; data source: estimation based on clinical experience) plus time to initiate eCPR after arrival in the emergency department (22 min; data source: mean time intervals as per the eCPR centre in Linz/Austria).

Using the estimated time from collapse to on scene eCPR (scenario 1) and the estimated time from collapse to in-hospital eCPR (scenario 2), the percentage of study patients, who would undergo eCPR within ≤30–≤75 min in five minutes intervals were calculated for both hypothetical scenarios. Based on publications on the duration of cardiopulmonary resuscitation before eCPR and its relationship with outcome,^12^, ^13^, ^14^, ^15^, ^16^ the estimated time to eCPR was categorised as short (≤45 min), intermediate (≤60 min) or too late (≤75 min).

### Definitions

We defined sustained ROSC in line with the Utstein criteria as the restoration of palpable central pulses and an autonomous ECG rhythm, which lasted for at least 20 min after the cessation of cardiopulmonary resuscitation.^24^

### Data processing

Only relevant sections of the medical records of the electronic database were transmitted by the OEAMTC Air Rescue service, strictly complying with both pseudo-anonymization and data minimization requirements. Following extraction of study variables from the electronic database, quality control cheques were performed to identify syntax or entry errors. Wherever possible, these errors were rectified. No data imputation methods were applied in case of missing values.

### Statistical analysis

All statistical analyses were performed with the SPSS software package (SPSS 30.0.0; IBM, Armonk, New York). Descriptive methods were used to report the characteristics of the study population as well as primary and secondary endpoints. Ninety-five percent confidence intervals (95 %CI) were calculated to report the precision of all study endpoints. Shapiro Wilks tests were applied to assess whether continuous variables approximately fulfilled normality assumption. Continuous variables are presented as mean values ± standard deviations, and categorical data as absolute numbers with percentages. In study patients, who fulfilled all eCPR eligibility criteria, the estimated time intervals until hypothetical eCPR were compared between the two scenarios using a Student’s *t*-test. A *p*-value < 0.05 was assumed to indicate statistical significance. The proportions of study patients, who fulfilled all eCPR eligibility criteria and hypothetically underwent eCPR within the ten predefined time periods, were compared between the two scenarios using Chi^2^-tests. In view of the fact that ten comparisons were made, the significance level for these comparisons was adjusted to a *p*-value < 0.005.

## Results

**Fig. 2:**
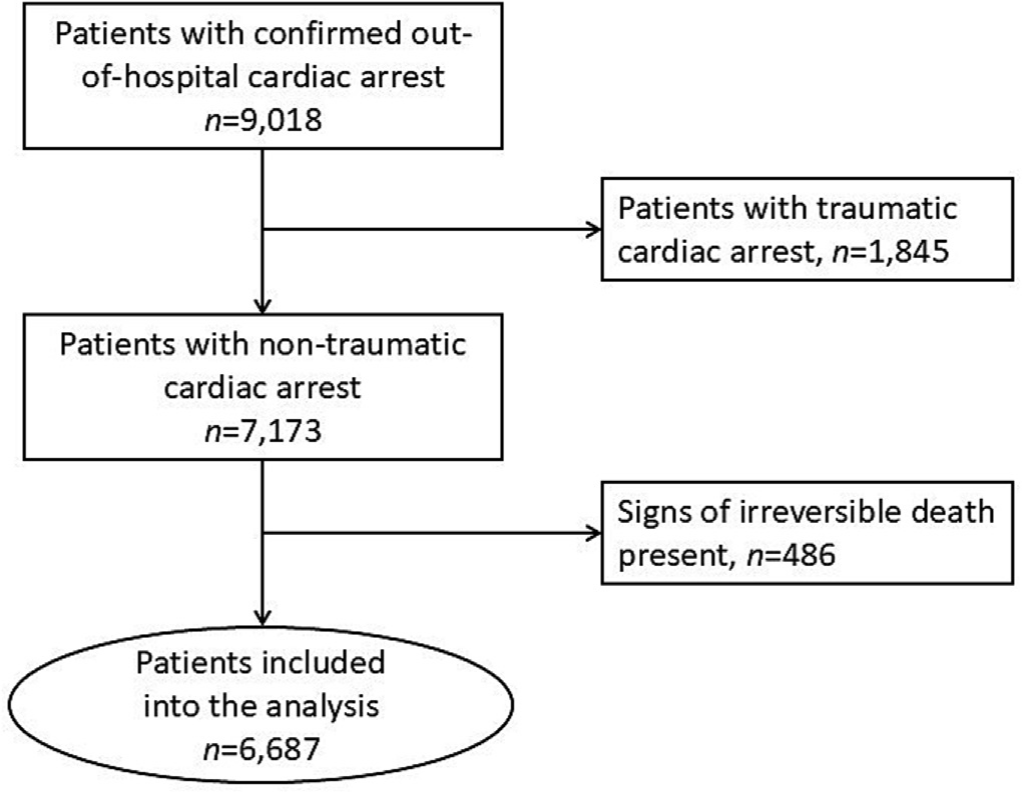
Study flow diagram.

Of 9018 patients with confirmed out-of-hospital cardiac arrest, 7173 patients were classified as having a non-traumatic cardiac arrest. Of these, 486 patients exhibited signs of irreversible death and were excluded. Six-thousand-six-hundred-eighty-seven patients were included into the statistical analysis (Fig. 2, Table 1).

**Table 1:** Characteristics of the study population and HEMS mission-related times.

***n***		6687
**Age**	years	67.7 ± 16.4
**Age groups**	*n* (%)	
*0 years*		46 (0.7)
*1*–*5 years*		44 (0.7)
*6*–*11 years*		16 (0.2)
*12*–*17 years*		17 (0.3)
*18*–*64 years*		2353 (35.2)
*≥65 years*		4211 (63.0)
**Male sex**	*n* (%)	4671 (69.9)
**Location where cardiac arrest occurred**	*n* (%)	
*Home*		2293 (34.3)
*Public space*		506 (7.6)
*Sport/outdoor*		432 (6.5)
*Workplace*		241 (3.6)
*EMS transport*		66 (1.0)
*Assisted living/nursing home*		52 (0.8)
*Other*		437 (6.5)
*Information missing*		2660 (39.8)
**Initial ECG rhythm**	*n* (%)	
*Asystole*		2581 (38.6)
*Ventricular fibrillation*		1149 (17.2)
*Shockable (AED)*		777 (11.6)
*Pulseless electrical activity*		766 (11.5)
*Non-shockable (AED)*		397 (5.9)
*Pulseless ventricular tachycardia*		56 (0.8)
*Information missing*		961 (14.4)
**Time from alarm to landing of HEMS team close to scene**	*min*	12.6 ± 4.7
**HEMS team first advanced critical care time on scene**	*n* (%)	5520 (82.5)
**Time of HEMS team on scene**	*min*	33.9 ± 19.1
**Sustained ROSC**	*n* (%)	2652 (39.7)
HEMS flight time to hospital*	*min*	10.5 ± 5.7

AED, automatic external defibrillator; CPR, cardiopulmonary resuscitation; ECG, electrocardiography; EMS, emergency medical service; HEMS, helicopter medical emergency system.

Data are given as mean values ± standard deviations, if not otherwise indicated.

* Only in patients with sustained ROSC.

All eCPR eligibility criteria were fulfilled in 890 [13.3 % (95 %CI, 12.5–14.2 %)] of the 6687 study patients (Table 2). This proportion varied when single eCPR eligibility were modified (Table 2). Sensitivity analyses yielded a proportion of 11.2 % (95 %CI, 10.4–11.9 %) if only patients aged 12–65 years or only those with an initial shockable ECG rhythm were considered eligible for eCPR. If patients were considered eligible for eCPR independent of the initial ECG rhythm the respective proportion was highest [21.6 % (95 %CI, 20.6–22.6 %)].

**Table 2:** Proportions of single and all eCPR eligibility criteria in the study population.

***n***		6687
**Single eCPR eligibility criteria fulfilled**		
*Age ≥12 and <70 years*	*n* (%)	3172 (47.4)
*Collapse witnessed by bystanders or EMS*	*n* (%)	3971 (59.4)
*Bystander or EMS CPR*	*n* (%)	4078 (61.0)
*Shockable ECG rhythm or PEA*	*n* (%)	2748 (41.1)
*No ROSC on HEMS arrival*	*n* (%)	6474 (96.8)
**All eCPR eligibility criteria fulfilled**	*n* (%)	**890 (13.3)**

**Sensitivity Analyses**		
**Sensitivity Analysis 1**		
*Changed criterion: Age ≥12 and <65 years*	*n* (%)	2521 (37.7)
All eCPR eligibility criteria fulfilled	*n* (%)	746 (11.2)

**Sensitivity Analysis 2**		
*Changed criterion: Only Shockable ECG rhythm*	*n* (%)	1982 (29.6)
All eCPR eligibility criteria fulfilled	*n* (%)	747 (11.2)

**Sensitivity Analysis 3**		
*Changed criterion: All ECG rhythms*	*n* (%)	6687 (100.0)
All eCPR eligibility criteria fulfilled	*n* (%)	1444 (21.6)

CPR, cardiopulmonary resuscitation; ECG, electrocardiography; eCPR, extracorporeal cardiopulmonary resuscitation; EMS, emergency medical services; HEMS, helicopter emergency medical service; PEA, pulseless electrical activity; ROSC, return of spontaneous circulation.

**Fig. 3:**
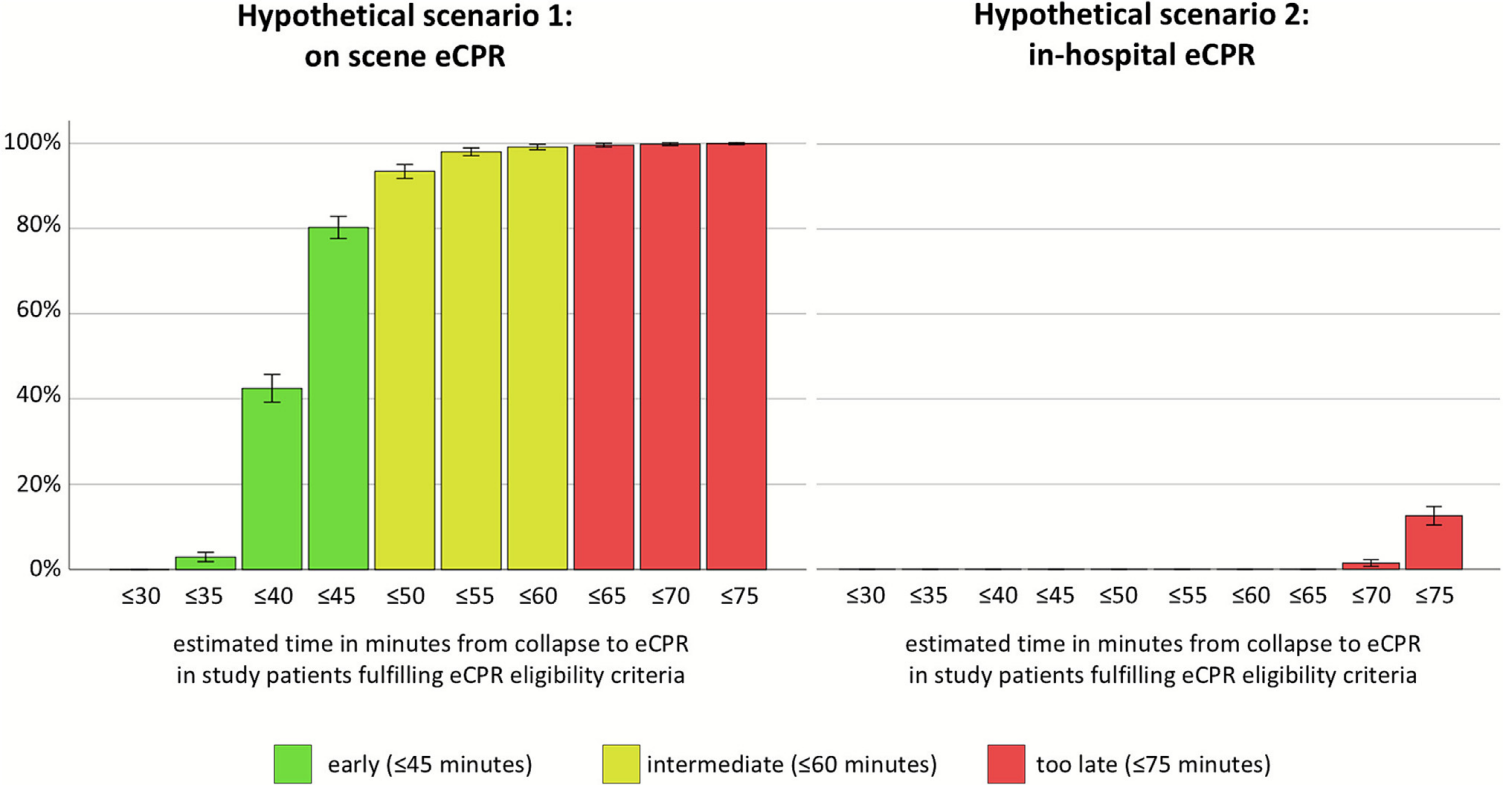
Proportions and 95 % confidence intervals of study patients, who fulfilled eCPR eligibility criteria on scene and underwent hypothetical eCPR within predefined time intervals for scenarios 1 and 2. eCPR, extracorporeal cardiopulmonary resuscitation.

**Supplementary Fig. E1:**
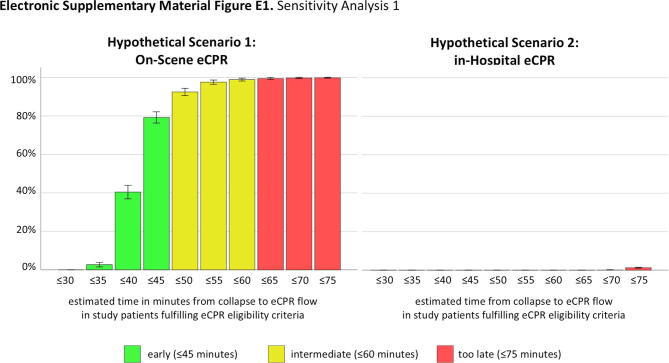


**Supplementary Fig. E2:**
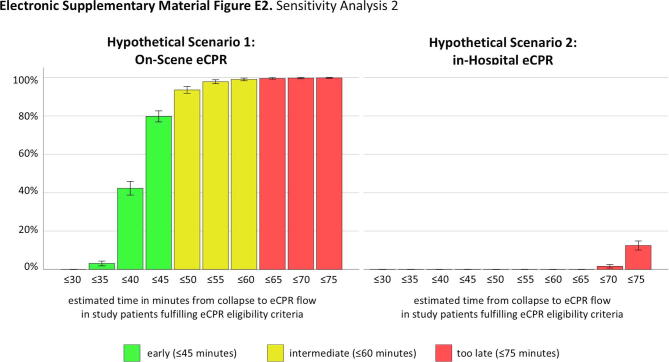


In patients, who fulfilled all eCPR criteria, the estimated time from collapse to hypothetical eCPR was significantly shorter in the on scene eCPR scenario than the in-hospital eCPR scenario (42.2 ± 5.2 vs. 82.4 ± 6.1 min, *p* < 0.001). Similar results were found for patients with different eCPR eligibility criteria (sensitivity analysis 1: 42.5 ± 5.3 vs. 82.6 ± 6.2 min, *p* < 0.001; sensitivity analysis 2: 42.3 ± 5.2 vs. 82.5 ± 6.2 min, *p* < 0.001; sensitivity analysis 3: 42.5 ± 5.4 vs. 83.3 ± 6.4 min, *p* < 0.001). Significantly more patients underwent hypothetical eCPR within 35, 40, 45, 50, 55, 60, 65, 70 and 75 min after collapse in scenario 1 (on scene eCPR) compared to scenario 2 (in-hospital eCPR) (*p* < 0.001 for all comparisons) (Fig. 3). No patient in scenario 1 nor scenario 2 would have achieved hypothetical eCPR flow within 30 min following collapse. Similar results were found in all sensitivity analyses (Electronic Supplementary Material Figs. E1–E3). The proportions of patients, who fulfilled eCPR eligibility criteria on scene, but could not undergo hypothetical eCPR within 60 min were significantly lower in scenario 1 (on scene eCPR) than in scenario 2 (in-hospital eCPR) [0.9 % (95 %CI, 0.4–1.8 %) vs. 100 % (95 %CI, 99.6–100 %), *p* < 0.001].

## Discussion

In this multicentre, retrospective cohort study, 13.8 % (95 %CI, 12.5–14.2 %) of all OHCA patients without signs of irreversible death fulfilled eCPR criteria at HEMS team arrival on scene. We acknowledge that different eCPR criteria have been published.^5^ In the present analysis, we focused on the latest recommendations of the European Resuscitation Council^6^ and the basic set of eCPR criteria as suggested by the Extracorporeal Life Support Organisation.^7^ In addition, we performed three sensitivity analyses which found similar results ranging between 11.2 (95 %CI, 10.4–11.9)% and 21.6 (95 %CI, 20.6–22.6)% of all OHCA patients. It is possible that the proportion of OHCA patients eligible for eCPR therapy would have been lower, if stricter (*e.g.*, age <60 years) or additional criteria (*e.g.*, endtidal carbon dioxide tension, near infrared spectroscopy) had been applied.

A prior study from Canada reported that an almost identical proportion of OHCA patients fulfilled eCPR eligibility criteria [14 % (95 %CI, 11–17 %)].^9^ While applying similar criteria in regards of age, witnessed collapse, and initial ECG rhythm, the authors accepted a liberal no flow time of <10 min, but included an expected transport time to the eCPR centre <20 min into their eCPR eligibility criteria.^9^ In another rather small study from Australia, 33 % of OHCA patients aged 18–65 years were considered eligible for eCPR.^11^ Two other studies, however, reported eCPR eligibility proportions among OHCA patients as low as 3.76 %^8^ and 4 %,^25^ respectively. One reason for the rather high proportion of eCPR eligible OHCA patients in our study could be the fact that criteria were applied at arrival of the HEMS team at the scene without taking delays due to expected transportation times into account. Given that only seventeen subjects in our cohort were aged between twelve and seventeen years, it is unlikely that lowering the age limit from eighteen to twelve years has relevantly contributed to the rather high proportion of eCPR eligible OHCA patients in our cohort.

The estimated time from collapse until hypothetical eCPR was significantly shorter in the on scene eCPR scenario than the in-hospital eCPR scenario. For this retrospective study, we used measured, published^22^, ^23^, ^26^ and estimated time intervals to assess the duration from collapse to hypothetical eCPR. This might have resulted in imprecise reporting of secondary study endpoints, particularly for study patients hypothetically undergoing intra-arrest transport and in-hospital eCPR. Notably, these estimated times (82.4 ± 6.1 min) corresponded well with median time intervals from collapse to eCPR data as reported by previous studies on in-hospital eCPR (74 min^27^; 75 min^28^; 76 min^29^). Importantly, the majority of patients in these studies were transported to eCPR centres by ambulance and not by HEMS, explaining the slightly shorter time intervals observed compared to our estimations. In line with this, a retrospective analysis from the United Kingdom reported an average time from 999 call to hospital arrival of OHCA patients under ongoing cardiopulmonary resuscitation of 86 min.^10^

Although not surprising, it was a striking result of our analysis that on scene eCPR flow within 60 min of collapse could have hypothetically been established in 99.1 % (95 %CI, 98.2–99.6 %) of OHCA patients eligible for eCPR, while not a single patient [0 %; 95 %CI, 0–0.4 %) would have hypothetically received in-hospital eCPR within this time window. This is particularly relevant as 60 min following collapse is the recommended interval during which eCPR should be initiated as recommended by current guidelines.^6^, ^7^ Taking these findings into account, one may conclude that it is highly unlikely that OHCA patients, who fulfil eCPR criteria and are managed by HEMS in Austria, would benefit from systematic intra-arrest transportation for in-hospital eCPR. Accordingly, the authors of a retrospective study conducted at an Austrian eCPR centre found that delays in collapse to eCPR times primarily occurred on scene and that it might be required that eCPR is taken to the patient rather than the patient to the hospital for eCPR.^29^

It is possible that the estimated proportion of eCPR eligible OHCA patients undergoing hypothetical in-hospital eCPR between 60 and 75 min was even overestimated in our cohort. This is due to the fact that we used the documented mean flight time of study patients who achieved a sustained ROSC to the hospital but not to the next eCPR centre. As eCPR is currently offered only at selected centres in Austria, it is likely that the actual intra-arrest transport times to eCPR capable hospitals would even be longer and the true proportions of eCRP eligible OHCA patients reaching one of these centres in time even lower. In addition, as indicated in previous studies,^11^, ^22^ some patients may achieve a stable ROSC during transportation further reducing the proportion of patients qualifying for eCPR after arrival in the emergency department.

Our results are in line with studies reporting significantly shorter time intervals until establishing extracorporeal blood flow, if eCPR therapy was initiated on the scene of OHCA compared to in-hospital eCPR.^28^, ^30^ The observation of a steep decrease in the proportion of eCPR eligible OHCA patients at hospital arrival when compared to the scene has previously been highlighted by a large US geographic information system model study.^8^ A smaller study from the United Kingdom including 162 eCPR eligible OHCA patients came to a very similar conclusion that in-hospital eCPR was of limited value for OHCA patients managed by HEMS teams in a semi-rural setting, even when dedicated pathways were in place.^10^ Similarly, a study from the Netherlands concluded that even in a healthcare system with relatively short transportation distances to hospital, consideration should be given to on scene eCPR for OHCA, as it shortens low-flow time and increases the number of potentially eligible patients.^22^

One key assumption underlying our analysis is that provision of eCPR by HEMS teams is both feasible and safe. It further implies that HEMS teams can constantly carry eCPR systems together with their medical equipment. As both weight and cabin space are crucial factors in HEMS, the question whether additional medical staff is required for effective eCPR delivery by HEMS teams needs to be addressed. Finally, helicopter transport of eCPR patients on extracorporeal circulation could pose a challenge both logistically and for patient safety. So far, mobile eCPR teams were initiated in several countries using car-based services.^17^, ^31^, ^32^ Recently, HEMS-based eCPR has been implemented in selected systems reporting feasibility and safety of such a setup.^33^, ^34^ Compared to car-based mobile eCPR services, HEMS-based eCPR has the potential to improve equity of eCPR access for eligible patients experiencing OHCA distant from eCPR centres. Trials are currently ongoing to elucidate the effects of HEMS-based eCPR on patient outcomes as well as the cost-effectiveness of such an intervention.^18^

Our study has several strengths including its large sample size. In addition, we analysed data of OHCA patients managed by the teams of thirteen out of sixteen bases of Austria’s largest HEMS provider. This allows extrapolation of our results to other central European environments with similar HEMS systems. On the other hand, important limitations need to be considered when interpreting our results. First, it was a retrospective analysis including patients over a period of ten years. According shortcomings such as incomplete or missing data (*e.g.*, the initial ECG rhythm was missing in 14.4 % of subjects), potential data misclassifications, and time-related confounders, may have occurred and potentially decrease the validity of our results. Second, three of the time intervals included into our calculations, specifically to describe the hypothetical scenario of in-hospital eCPR therapy, were estimated as these were neither measured nor documented in the database. Although we used published time intervals, mean values of established eCPR systems or rather conservative estimations based on the authors’ clinical experience, these intervals may have still deviated from actual times. Finally, Austrian HEMS largely operates in semi-rural, rural or alpine rather than urban settings. Geographic modelling studies demonstrated that urban areas have a higher proportion of eCPR eligible OHCA patients compared to rural settings, largely because of shorter response and transport times of emergency medical services allowing more patients to meet eCPR eligibility criteria.^8^ In this context, it is important to note that data from the HEMS base in Vienna, the largest metropolitan region in Austria and also home to the largest eCPR centre of Austria,^29^ were not included into this study.

## Conclusions

In Austrian HEMS, one in seven patients with OHCA fulfilled eCPR eligibility criteria on scene. Our estimates strongly suggest that initiation of eCPR on scene could significantly shorten low-flow times and increase the proportion of eligible OHCA patients, who receive this potentially life-saving intervention.

## Funding sources

The research did not receive any specific grant from funding agencies in the public, commercial, or not-for-profit sectors.

## CRediT authorship contribution statement

**Romana Erblich:** Writing – review & editing, Visualization, Validation, Supervision, Methodology, Data curation, Conceptualization. **Matthias Noitz:** Writing – review & editing, Data curation. **Helmut Trimmel:** Writing – review & editing, Supervision, Methodology, Conceptualization. **Wolfgang Voelckel:** Writing – review & editing, Supervision, Methodology, Conceptualization. **Dinis Dos Reis Miranda:** Writing – review & editing, Methodology. **Dominik Jenny:** Writing – review & editing, Data curation. **Oliver Hunsicker:** Writing – review & editing, Visualization, Methodology. **Maximilian Havlicek:** Writing – review & editing, Data curation. **Julian M. Baumkirchner:** Writing – review & editing, Data curation. **Jens Meier:** Writing – review & editing, Validation, Supervision, Methodology, Formal analysis. **Martin W. Dünser:** Writing – original draft, Visualization, Validation, Supervision, Methodology, Formal analysis, Conceptualization.

## Declaration of competing interest

The authors declare that they have no known competing financial interests or personal relationships that could have appeared to influence the work reported in this paper.

## Figures and Tables

**Supplementary Fig. E3:**